# The genus *Sternocampsus* Fleutiaux, 1927 (Coleoptera, Elateridae, Oxynopterinae), with description of a new species from South China

**DOI:** 10.3897/zookeys.852.31611

**Published:** 2019-06-05

**Authors:** Zhen Liu, Shi-hong Jiang

**Affiliations:** 1 School of Applied Chemistry and Biological Technology, Postdoctoral Innovation Practice Base, Shenzhen Polytechnic, Shenzhen, Guangdong 518055, China Shenzhen Polytechnic Shenzhen China; 2 College of Life and Environmental Science, Hunan university of Arts and Science, Changde, 41500, China Hunan university of Arts and Science Changde China

**Keywords:** China, distribution, Elateroidea, key, new taxon, new combination, taxonomy

## Abstract

*Sternocampsuscoriaceus*, **sp. nov.** is described and illustrated from China. A new combination, *Campsosternuscastaneus* (Jiang & Wang, 1999) is proposed. A key and a checklist of the known species, together with a distribution map of Chinese *Sternocampsus* species, are provided.

## Introduction

The Oriental genus *Sternocampsus* Fleutiaux, 1927 (Coleoptera: Elateridae) was established for a single species, viz. *S.villosus* Fleutiaux, 1927, from Pahang, Malaysia. Although these *Sternocampsus* are large-bodied beetles, specimens are rarely collected. After 72 years, a second species, *S.castaneus* Jiang, 1999, was discovered in Yunnan, China (Jiang and Wang 1999). However, we propose to transfer it to *Campsosternus* for reasons below.

*Sternocampsus* belongs to the subfamily Oxynopterinae Candèze, 1857 and morphologically resembles *Oxynopterus* Hope, 1842, the monotypic genus *Sinuaria* Jordans, 1894 and *Campsosternus* Latreille, 1834. *Sinuaria* can be easily distinguished from *Sternocampsus* by the strongly sinuate pronotal edges, strongly retracted head and non-serrate antenna. Flabellate antennae are diagnostic for *Oxynopterus*, however antennae are serrate in *Sternocampsus* and *Campsosternus*. *Sternocampsus* is also like *Campsosternus* in having a prominent mesoventrite and the male aedeagus has parameres with hook-like apices. But *Sternocampsus* differs from *Campsosternus* by the following (*Campsosternus* characteristics in parentheses): having a smaller and a somewhat retracted head (larger and less retracted); the pronotum is flat from lateral view and wider across the hind angles than the median length (often evenly convex and usually as wide as long); hind angles long and strongly divergent (short and less divergent); the prosternum is concave sublaterally (often flat or slightly concave); elytra without striae (often with striae); and the body dark without a metallic sheen (often with metallic sheen) (Latreille 1834; Fleutiaux 1927; Kishii 1987).

Here, we describe a new species of *Sternocampsus* from South China and propose a new combination (i.e., *Campsosternuscastaneus* (Jiang & Wang, 1999), **comb. nov**.). The new species is illustrated along with a key to the two known species of *Sternocampsus*.

## Material and methods

Studied specimens belong to the following collections:

**BMNH**, British Museum of Natural History, London, UK

**MHBU**, the Museum of Hebei University, Baoding, China

**MNHN**, Muséum National d’Histoire Naturelle, Paris, France

**SNUC**, Insect Collection of the Shanghai Normal University, Shanghai, China

**SWU**, Institute of Entomology, Southwest University, Chongqing, China

**SZPT**, School of Applied Chemistry and Biological Technology, Shenzhen Polytechnic, Shenzhen, Guangdong Province, China.

The terminology used mainly follows Costa et al. (2010) and Douglas (2011). The classification follows Cate et al. (2007). Observations and measurements were made under a stereomicroscope Motic SMZ-168. Photographs were made using a digital microscope (LY-WN-YH 3D system), Canon EOS-1 camera with Canon EF 100 mm, 65 mm and 55–250 mm lens.

Measurements: body length was measured along the midline from the anterior edge of the head capsule to the apex of elytra; body width was measured across the broadest part (usually across the elytra). Pronotal length was measured along the midline; the pronotal width was measured across the broadest part (usually across the hind angles).

Specimens were mounted on paper cards. The genitalia were removed, cleaned and fixed under the body of the specimen in glycerol mounts following Prosvirov and Savitsky (2011).

## Taxonomy

### Campsosternus castaneus

Taxon classificationAnimaliaColeopteraElateridae

(Jiang & Wang, 1999) comb.nov.

**Figure 1.**
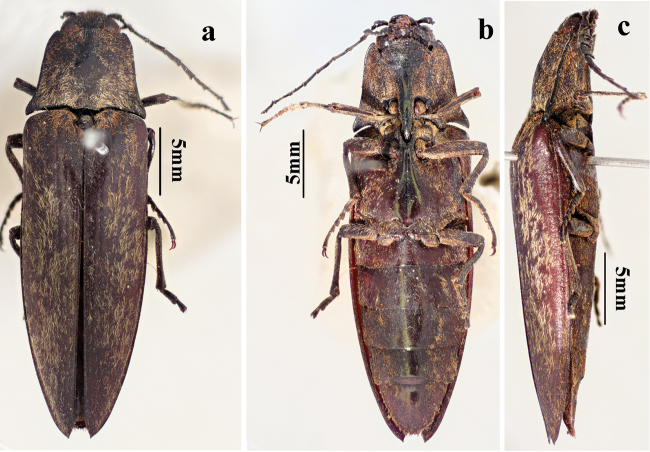
Habitus of *Campsosternuscastaneus* (Jiang & Wang, 1999). comb. nov., holotype, male: **a** dorsal view **b** ventral view **c** lateral view.

**Figure 2.**
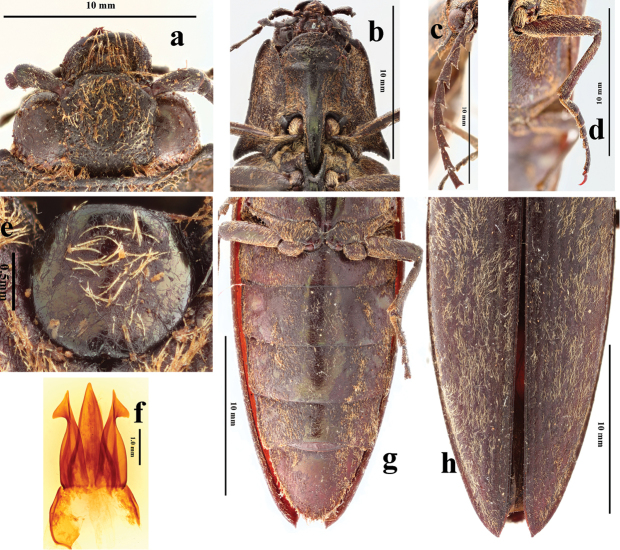
*Campsosternuscastaneus* (Jiang & Wang, 1999). comb. nov., holotype, male: **a** head, dorsal view **b** prothorax, ventral view **c** antennomeres 1–10, dorsal view **d** middle leg, ventral view **e** scutellar shield, dorsal view **f** aedeagus **g** abdomen, ventral view **h** elytra, dorsal view.

**Figure 3.**
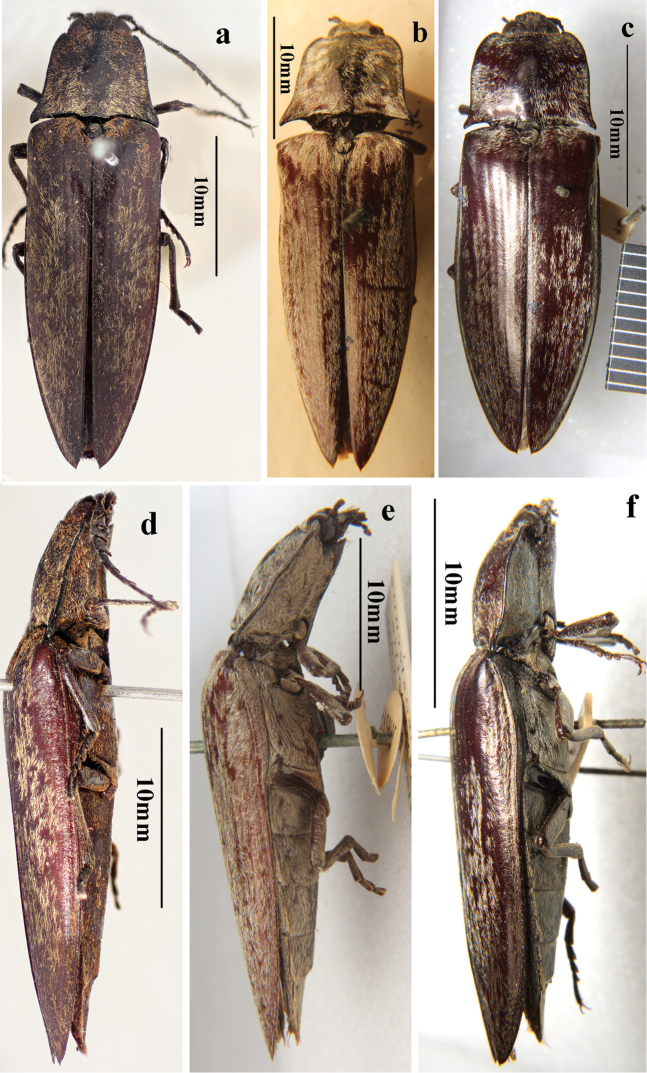
Habitus of *Campsosternus* spp. (all BMNH, photos by Dr. Yong-ying Ruan): **a–c** Dorsal view of *Campsosternus* spp. **a***C.castaneus* (Jiang & Wang, 1999). comb. nov. **b***C.argentipilis* (Candéze, 1874) **c***C.saundersi* (Candéze, 1874) **d–e** Lateral view of *Campsosternus* spp. **d***C.castaneus* (Jiang & Wang, 1999). comb. nov. **e***C.argentipilis* (Candéze, 1874) **f***C.saundersi* (Candéze, 1874).

Figs 1a–c , 2a–h , 3a, d

Sternocampsus castaneus : Jiang and Wang 1999: 34; Jiang 1993: 136 [*nomen nudum*].

#### Material examined.

Holotype of *C.castaneus*, China (SZPT). ♂, “Mengla (650m), Yunnan, 1982. IV.12, Jin Gentao, No. 0545”.

Syntype (images) of *Campsosternusargentipilis* (Candéze, 1874) (BMNH), label 1 “type”, label 2 “Siam”, label 3 “*Campsosternus argentipilis* Type Cdze”.

Syntype (images) of *C.saundersi* (Candéze, 1874) (BMNH), label 1 “type”, label 2 “Siam Laos”, label 3 “Laos Mouhot”, label 4 “*Campsosternus saundersii* Cdze Type ex coll. Saunders”, label 5 “*Campsosternus saundersii* type”, label 6 “Jancon coll. 1903-130.”, label 7 “BMNH (E) #1024842”.

#### Diagnostic note.

Based on the original description, this species has: body length 30.5–32.0 mm, elongate, chestnut brown integument, covered with silvery white pubescence (Fig. 1a–c). Antenna (Fig. 2c) reaching beyond apex of hind angle of pronotum by length of four apical antennomeres. Pronotum 1.2 times wider than long, slightly convex, with punctures medially, spaces between punctures 5 puncture diameters wide, nearly impunctate laterally; hind angles long, acute, divergent, with a distinct carina. Scutellar shield (Fig. 2e) 1.1 times wider than long, with punctures, spaces between punctures 5 to 8 puncture diameters wide. Elytra wider than prothorax, 3.5 times longer than prothorax; convex with micro-striae, interstrial punctures sparse and smallest of all punctures on body; interstriae flat with very shallow punctures, irregularly distributed, faintly transversally rugose. Penis (Fig. 2f) narrowed to acute with sides convex near base, straight near apex, parameres with longitudinal carina, hook-like at apex.

#### Notes.

Based on examination of the types this species should be transferred from genus *Sternocampsus* to *Campsosternus* because of its convex and nearly square-shaped pronotum (anterior quarter 2/3 width of hind angles), the larger protruding head (less than half of eyes hidden by thorax in dorsal view) and the striate elytra. This species is similar to several dark colored *Campsosternus* species (cf. Fig. 3b, e: *C.argentipilis* (Candéze, 1874) and Fig. 3c, f: *C.saundersi* (Candéze, 1874)). They share several characters: prominent, large head with only slightly protruding frons, long pubescence, striate elytra. Furthermore, the green metallic sheen is somewhat present on the scutellum and medio-longitudinal area of the ventral part of *C.castaneus* (Figs 1b, 2e), shared with most *Campsosternus* spp. *Campsosternuscastaneus* also differs from *C.argentipilis* by the density of the pubescence, punctures on pronotum and elytra, and body size, and is distinguished from *C.saundersi* by the shape of the pronotum, body size and ratios.

#### Distribution.

China (Yunnan).

### Sternocampsus

Taxon classificationAnimaliaColeopteraElateridae

Fleutiaux, 1927

Sternocampsus Fleutiaux, 1927: 104; Jiang 1993: 136; Jiang and Wang 1999: 34; Cate et al. 2007: 94.

#### Type species.

*Sternocampsusvillosus* Fleutiaux, 1927, by monotypy.

#### Diagnosis.

Head narrow compared to pronotum (ratio of head width between eyes to pronotum width across hind angles varied from 1/5 to 1/6). Frons flattened medially to level of labrum, frontal carina incomplete medially; mandibles arched, protruding. Antenna of both sexes, exceeding hind angles of pronotum, compressed, and serrate from 3^rd^ to 10^th^ antennomeres. Pronotum narrowed anteriorly, both sides distinctly sinuate, strongly flanged laterally, weakly convex medio-longitudinally; hind angles divergent, acute, apex recurved. Suture between meso- and metaventrite weak. Elytra attenuate, apically with a spine, surface almost smooth, with slightly irregular furrows posteriorly. Penis narrowed to apex, parameres with hook-like angles apically.

#### Distribution.

Malaysia (Pahang), China (Guangdong, Guangxi, Hunan, Fujian) (Fig. 9).

### Sternocampsus coriaceus

Taxon classificationAnimaliaColeopteraElateridae

Liu & Jiang sp. nov.

http://zoobank.org/E08008CE-DB51-49F6-85C5-5929891CD826

**Figure 4.**
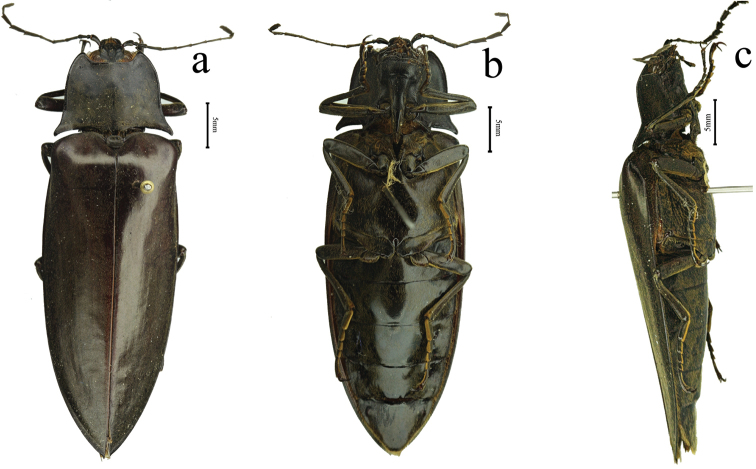
Habitus of *Sternocampsuscoriaceus* sp. nov., holotype, male: **a** dorsal view **b** ventral view **c** lateral view.

**Figure 5.**
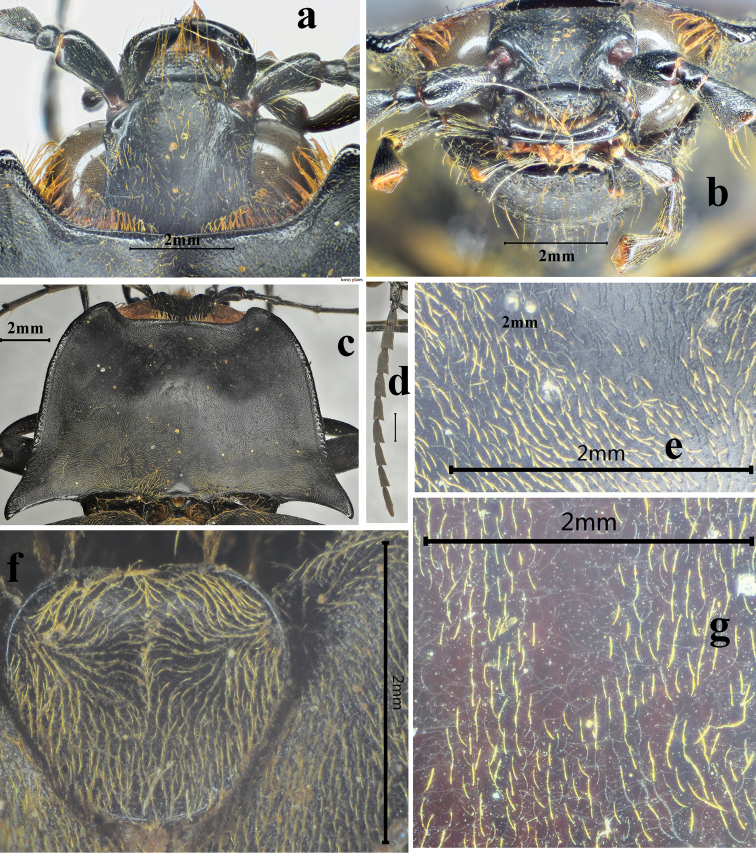
*Sternocampsuscoriaceus* sp. nov., holotype, male: **a** head, dorsal view **b** head, frontal view **c** pronotum, dorsal view **d** antenna, dorsal view **e** surface of pronotum, dorsal view **f** scutellar shield, dorsal view **g** surface of elytra, dorsal view.

**Figure 6.**
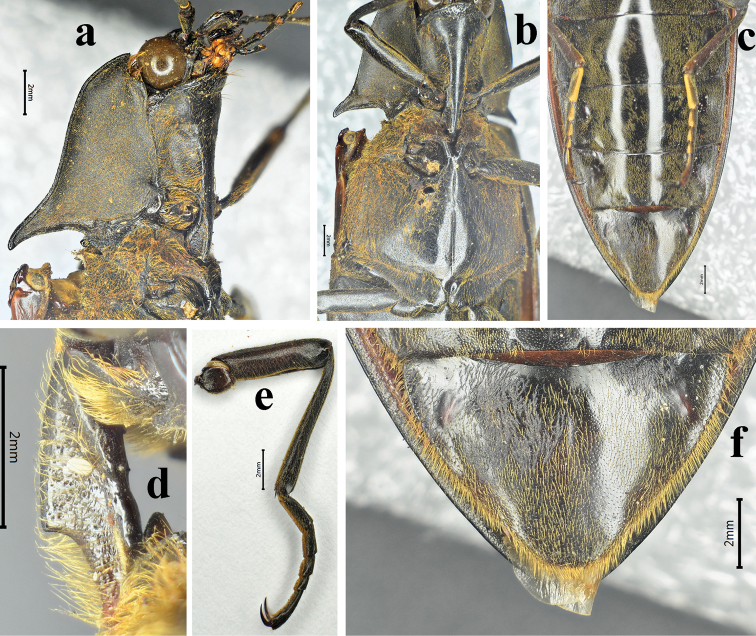
*Sternocampsuscoriaceus* sp. nov., holotype, male: **a** prothorax, lateral view **b** thorax, ventral view **c** abdomen, ventral view **d** prosternal process, lateral view **e** middle leg, lateral view **f** sternite VII, ventral view.

**Figure 7.**
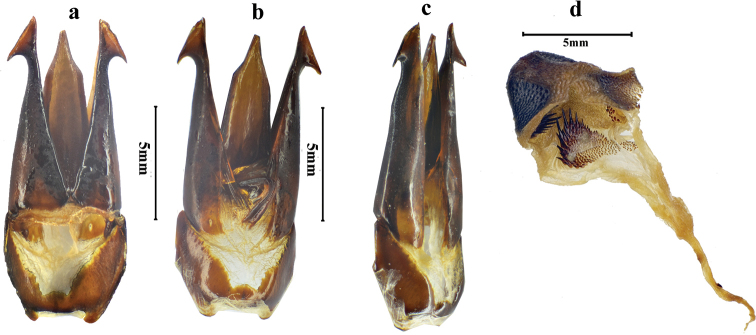
*Sternocampsuscoriaceus* sp. nov., **a–c** dorsal, ventral and lateral view of aedeagus **d** bursa copulatrix and thorny plates.

**Figure 8.**
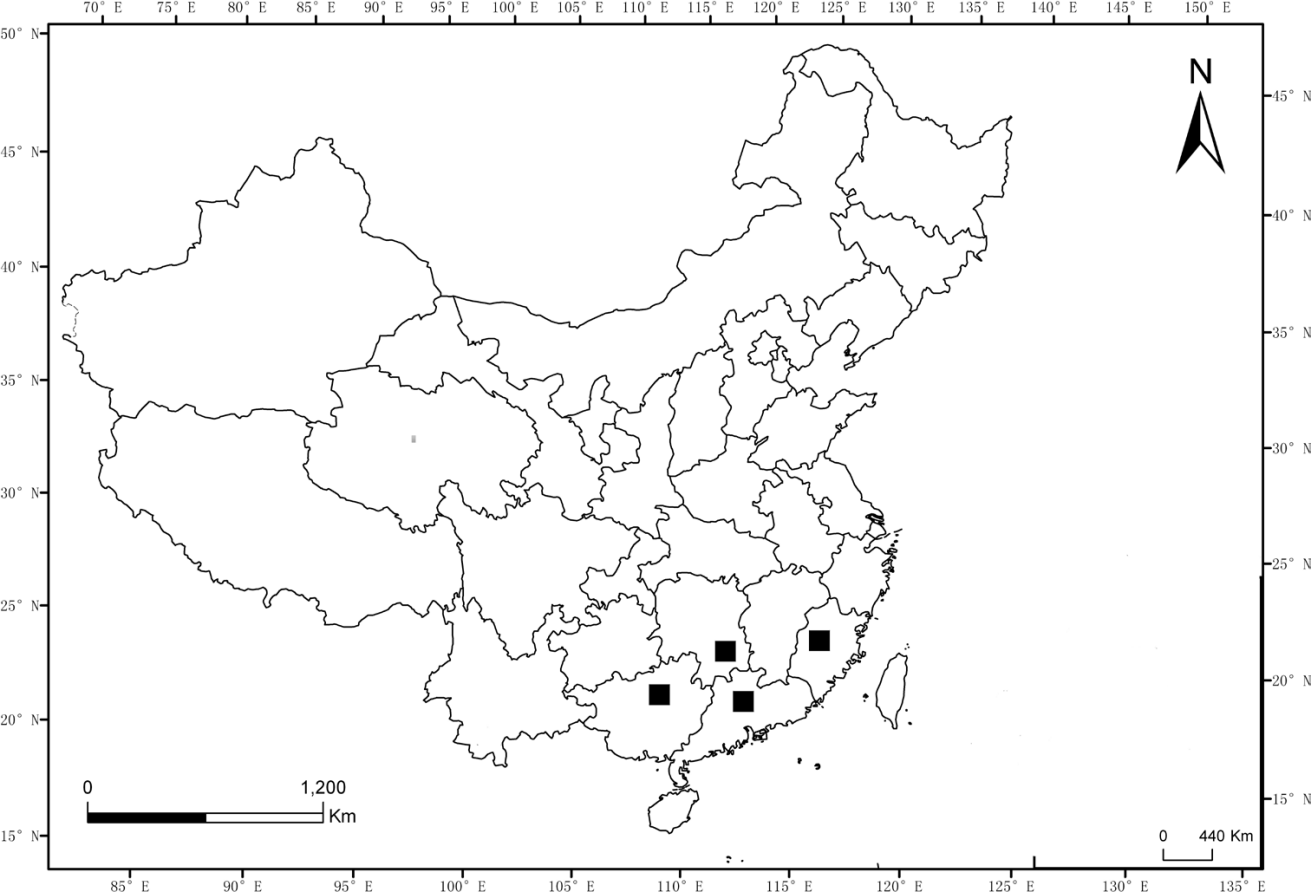
Distribution map of genus *Sternocampsus* in China. ■ *S.coriaceus* sp. nov.

Figs 4a–c , 5a–g , 6a–f , 7a–d , 8

#### Material examined.

Holotype. ♂, Guangdong Prov., Nanling Natural Reserve, 12.V.2001, Ming-yi Tian, No. 20180380 (SZPT). Paratypes: 1♀, Guangdong Prov., Nanling Natural Reserve, VI–VII.2001, Lei Gao, No. 20180381 (SZPT); 2♀♀7♂♂, Guangdong prov., Nanling Natural Reserve, VII–VIII,2011, Bei-kun Chen, Nos. 20180384, 20180385, 20180386, 20180387, 20180388, 20180389, 20180390, 20180391, 20180392 (SZPT); 6♂♂, Guangdong prov., Nanling Natural Reserve, V.2001, Ming-yi Tian, Nos. 20180393, 20180394, 20180395, 20180396, 20180397, 20180398 (SZPT); 1♂, Guangdong Prov. (light trap), Nanling Natural Reserve, 11.V. 2009, Ding Chen, No. 20180399 (SZPT); 1♂, Guangdong prov. (light trap), Nanling Natural Reserve (1000m), 3.V.2004, Jin-cheng Zeng, No. 20180400 (SZPT); 1♂, Guangdong Prov., Nanling Natural Reserve, VI–VII.2008, Lei Gao No. 20180401 (SZPT); 1♂, Guangdong Prov. (light trap), Nanling Natural Reserve, VII.2008, Kai-xuan Chen, No. 20180402 (SZPT); 1♀, Guangdong Prov., Nanling Natural Reserve (Nanling protection station), V.2010, Chen-Hui Zhan, No. 20180405 (SWU); 3♂♂, Guangdong Prov., border between Nanling N.R. and Mangshan N.R., 5.V.2017, Jin-Kun Zhang, Nos. 20180406, 20180407, 20180408 (1 in SNUC, ex SWU; 2 in SWU); 1♀1♂, Guangxi Prov., Maoer Mts., 3.VII.2003, Min Wang, Nos. 20180382, 20180383 (SZPT); 1♂, Fujian prov., Wuyi Natural Reserve, 5–20.VII.2003, Ming Bai et Guo-dong Ren, No. 20180403 (MHBU); 1♂, Hunan Prov. (light trap, 1430m), Yizhang County, Mangshan Natural Reserve (Xiangsikeng), 2.VII.2017, Ren-Zhi Zhang (SNUC, ex SWU), No. 20180404.

#### Diagnosis.

Body not-metallic. Pronotum nearly twice wider (across hind angles) than its median length, flat in dorsal view, with four shallow depressions between longitudinal and transverse middle line. Elytra widest at apical third, shiny, smooth, coriaceous-rugulose sculpture hardly visible, without striae or linear punctures, covered with short pubescence, 1/5 length of diameter antennomere 2. Penis width measured before apical attenuation 3.3 times wider than minimum width of paramere, and penis not reaching beyond parameres.

#### Description.

**Male** (holotype). Body length 47.5 mm, width 14.5 mm. Body dark red-brown to black (Fig. 4a), nearly impunctate. Pronotum, head, ventral parts of body, antenna and legs dark brown to black, elytra dark red-brown, dark laterally, strongly shiny; dorsal pubescence orange, recumbent, and extremely short, denser on pronotum, ventral pubescence longer and denser.

*Head.* Head semi-retracted (concealing most of eyes in dorsal view in Fig. 5c), frons broadly depressed, smooth and impunctate medially, uneven with weak rugose punctures laterally, surface with sparse, short pale orange pubescence, frontal carina incomplete, only present above antennae, moderately protruding above rest of frons in lateral view. Eyes bulbous (Figs 5a, 6a). Mandible strongly incurved, nearly right angled, simple. Labrum semicircular, with dense rugose punctures laterally and long (length 1.8 times longer than diameter of antennomere 2) orange pubescence medially (Fig. 5a). Last segment of maxillary palpus truncate apically, triangular and 1.7 times longer than wide. Antenna (Fig. 5d) reaching beyond hind angles of pronotum, with long (1/3 length of diameter antennomere 2) orange pubescence, serrate from antennomeres 3 to 10, and gradually narrowing from antennomere 4^th^ onward; antennomere 1 clavate, elliptically concave dorsal-subapically, and 1.7 times longer than wide; antennomere 2 shortest, rounded, slightly longer than wide; antennomere 3 2.3 times longer than antennomere 2 and 0.6 times antennomere 4; antennomeres 4 to 10 elongate triangular, attached latero-apically to preceding antennomere; antennomere 11 4.7 times longer than its maximum width, and 1.4 times longer than antennomere 1, with a constriction at apical fourth.

*Thorax*. Pronotum nearly twice wider (across hind angles) than its length, impressed on either side of midline near anterior and posterior thirds (Fig. 5c), bordered by carina at sides and posterad. Disc polished, with short orange pubescence, longer and denser along hind margin; anterior angles broadly protruding anterad near head, pronotal setae long anteriorly to half covering eyes in Fig. 5a; hind angles long, and divergent, upheaved and acute, apices curved downwards, without dorsal carina. Anterior lobe of prosternum rugose-punctate (Fig. 6b). Prosternal sutures sinuate and not bordered by raised carina on hypomeraon; prosternum shiny with small punctures, spaces between punctures 2 to 3 puncture diameters wide and sparse pubescence, length 1/2 of diameter antennomere 2, surface sulcate laterally (Figs 4b, 6a, b); prosternal process straight in lateral view, acuminate in dorsal view, area between the dorsal and ventral apices (*sensu*Douglas 2011) concave, ventral surface shorter than dorsal surface (Fig. 6b, d). Meso- and metaventrite smooth with regular small punctures, covered with thick and orange pubescence, half length of diameter antennomere 2, and suture between ventrites shallow; metaventrite narrowly furrowed medially along entire length (Fig. 6b).

*Scutellar shield.* (Fig. 5f) Flat, widest in anterior third, straight anteriorly with rounded lateral corners and posterior end, broadly concave medially in dorsal view, nearly straight on posterior sides, slightly wider than long, punctate, pubescent.

*Elytra.* 4 times longer than and slightly wider than pronotum (measured across hind angles), elongate (Figure 4a), anterior two-thirds nearly parallel-sided narrowed at posterior third, each apex with spine; shiny, smooth, with fine coriaceous-rugulose sculpture, without striae or linear punctures, covered with pubescence, which 1/5 length of diameter antennomere 2 (Fig. 5g).

*Legs* (Fig. 6e) Covered with extremely dense and regular pubescence. Tarsi with yellow-brown bristle pad underneath, tarsomeres 1 to 4 becoming sequentially shorter, tarsomeres1 nearly equal to tarsomere 5, tarsomere 5 longest, tarsomere 4 shortest. Metacoxal plate (Fig. 6b) with mesal third nearly parallel-sided, then abruptly and strongly narrowed into a narrow strip laterally.

*Abdomen.* General surface like that of metaventrite. Sternite III–VII each with paired round red-brown tubercles laterally, sternite VII emarginate basal-medially, abruptly narrowed posterad, triangular in ventral view, sinuate laterally, with weak longitudinal snowflake-like rugosity near sides anterad (Fig. 6f).

*Genitalia.* Penis width measured before apical attenuation 3.3 times wider than minimum width of paramere, and penis slightly shorter than parameres, apex abruptly and strongly narrowed, with low thorny tubercles; parameres with incision near each base in ventral view, sides nearly straight, and then strongly concave towards apex, with pre-apical acute hook-like expansion (Fig. 7a–c).

**Female.** Like male, except longer (17.0–17.5 mm) and with shorter antennae. Bursa copulatrix with four symmetrical thorny plates inside (Fig. 7d).

#### Variations.

Body length: 45.5–51.0 mm (male) or 52.0–53.5 mm (female); body width: 13.5–17.0 mm (male) or 17.0–17.5 mm (female).

#### Larva.

Unknown.

#### Etymology.

The specific name “*coriaceus*” (Latin for “leather-like”) refers to the coriaceous sculpture of the elytra.

#### Distribution.

China: Guangdong (Nanling Natural Reserve), Guangxi (Maoer Mts.), Fujian (Wuyi Mts.), Hunan (Mangshan Natural Reserve) (Fig. 8).

#### Biology.

Unknown, but collected at light traps at night. Some specimens collected at the elevation of 1430 m in subtropical forest.

#### Remarks.

This species is the second species of the genus *Sternocampsus* Fleutiaux. It differs from the congener *S.villosus* Fleutiaux, 1927 by the following: smaller body (45.5–53.5 mm; 55 mm in *S.villosus*); antennomere 3 shorter than antennomere 1 (antennomere 3 longer than 1 in *S.villosus*); and pubescence of hypomeron sparser and shorter (thicker and longer in *S.villosus*). The shape and arrangement of thorny plates in female bursa copulatrix (Fig. 7d) of *S.coriaceus* differs from *Campsosternus* spp. (Hsieh et al. 2014)), which also supports diagnosis of genus *Sternocampsus* Fleutiaux.

### Sternocampsus villosus

Taxon classificationAnimaliaColeopteraElateridae

Fleutiaux, 1927

Fig. 9a–h

Sternocampsus villosus Fleutiaux, 1927: 104.

#### Material examined.

**Synt**ype (images) of *C.villosus*, ♂, (MNHN), label 1 “Malay Penins:/ Pahang F.M.S./Cameron Highland/ Tanah Rata/ Febr. 12^th^ 1926”, label 2 “Musee Kua Lumpur:/Penang/Collection FLEUTIAUX”, label 3 “*Sternocampsus villosus* ♂/ Fleut. Type/ Collection FLEUTIAUX”., label 4 “Collection E. Fleutiaux”, label 5 “TYPE”, label 6 “SYNTYPE”, label 7 “SYNTYPE *sternocampsus villosus* Fleutiaux, 1927”, label 8 “MNHN EC9700”. Syntype (images) of *C.villosus* (MNHN), label 1 “Pahang F.M.S./ ”Cameron’s Highlands”/ ?/ 4800 ft. Mars. ?. 1924/ H.M.Pendlebury.”, label 2 “Musee Kuala Lumpur:/ Penang/ Collection FLEUTIAUX”, label 3 “*Sternocampsus villosus*/ Fleut. cotype/ Collection FLEUTIAUX”., label 4 “Collection E. Fleutiaux”, label 5 “SYNTYPE”, label 6 “SYNTYPE *sternocampsus villosus* Fleutiaux, 1927”, label 7 “MNHN EC9701”.

#### Diagnosis.

According to the original description, this species is characterized by: body length 55 mm, large and robust; elytra not metallic, red-brown; pubescence yellow and thick. Frons depressed medially. Antenna black; similar in both sexes, but somewhat longer in male than in female; antennomere 1 longer than remaining antennomeres, widened apically; antennomere 2 very small; antennomere 3 longer than 1 and shorter than 4. Pronotum smooth. Elytra more or less dark brown, almost smooth, sculpture hardly discernable. Black ventrally, pubescence of hypomeron very thick. Legs black.

#### Notes.

**Figure 9.**
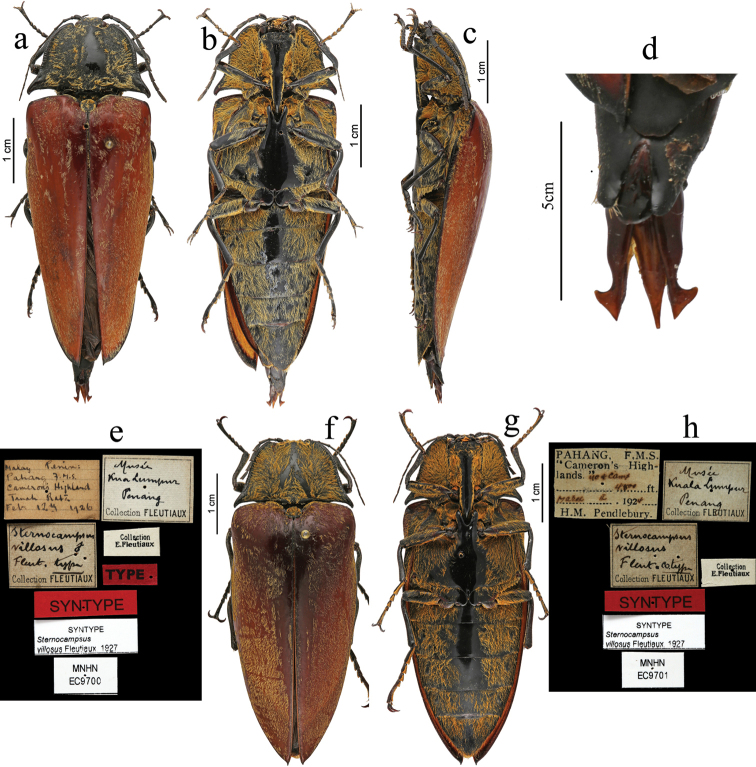
*Sternocampsusvillosus* Fleutiaux, 1927, **a–e** syntype, male: **a** dorsal view **b** ventral view **c** lateral view **d** aedeagus, dorsal view **e** labels of type **f–h** syntype, female: **f** dorsal view **g** ventral view **c** labels of syntype. (Copyright © Antoine Mantilleri. All rights reserved MNHN/A. Mantilleri).

We have checked the images of types from the MNHN taken by Dr. Antoine Mantilleri. Elytra were red-brown from the images (Fig. 9a, f), not dark brown as in the original description. From the image (Fig. 9d), the characters of its aedeagus (penis extending slightly beyond parameres, weakly sinuate before acute apex; the outer margin of parameres abruptly incurved before apical hook-like pre-apical expansions) differ from *S.coriaceus*.

#### Distribution.

Malaysia (Pahang).

### Key to species of genus *Sternocampsus* Fleutiaux worldwide

**Table:** 

1	Length of body 45.5–51.0 mm; elytra nearly black (Fig. 4a); parameres straight basad of pre-apical expansion, penis not extending beyond parameres	***S.coriaceus* sp. nov.**
–	Length of body 55 mm; elytra brown or red-brown (Fig. 9a, f); parameres sinuate basad of pre-apical expansion, penis extending beyond parameres	***S.villosus* Fleutiaux, 1927**

### Checklist of *Sternocampsus* species

*Sternocampsuscoriaceus* Liu & Jiang, sp. nov. [China (Guangdong, Guangxi, Hunan, Fujian)]

*Sternocampsusvillosus* Fleutiaux, 1927 [Malaysia (Pahang)]

## Supplementary Material

XML Treatment for Campsosternus castaneus

XML Treatment for Sternocampsus

XML Treatment for Sternocampsus coriaceus

XML Treatment for Sternocampsus villosus
